# Stable and Dynamic Coding for Working Memory in Primate Prefrontal Cortex

**DOI:** 10.1523/JNEUROSCI.3364-16.2017

**Published:** 2017-07-05

**Authors:** Eelke Spaak, Kei Watanabe, Shintaro Funahashi, Mark G. Stokes

**Affiliations:** ^1^Department of Experimental Psychology, University of Oxford, Oxford OX1 3UD, United Kingdom,; ^2^Center for Information and Neural Networks (CiNet), National Institute of Information and Communications Technology, Osaka 565-0871, Japan, and; ^3^Kokoro Research Center, Kyoto University, Kyoto 606-8501, Japan

**Keywords:** dynamic coding, memory-guided saccade, prefrontal cortex, representational geometry, working memory

## Abstract

Working memory (WM) provides the stability necessary for high-level cognition. Influential theories typically assume that WM depends on the persistence of stable neural representations, yet increasing evidence suggests that neural states are highly dynamic. Here we apply multivariate pattern analysis to explore the population dynamics in primate lateral prefrontal cortex (PFC) during three variants of the classic memory-guided saccade task (recorded in four animals). We observed the hallmark of dynamic population coding across key phases of a working memory task: sensory processing, memory encoding, and response execution. Throughout both these dynamic epochs and the memory delay period, however, the neural representational geometry remained stable. We identified two characteristics that jointly explain these dynamics: (1) time-varying changes in the subpopulation of neurons coding for task variables (i.e., dynamic subpopulations); and (2) time-varying selectivity within neurons (i.e., dynamic selectivity). These results indicate that even in a very simple memory-guided saccade task, PFC neurons display complex dynamics to support stable representations for WM.

**SIGNIFICANCE STATEMENT** Flexible, intelligent behavior requires the maintenance and manipulation of incoming information over various time spans. For short time spans, this faculty is labeled “working memory” (WM). Dominant models propose that WM is maintained by stable, persistent patterns of neural activity in prefrontal cortex (PFC). However, recent evidence suggests that neural activity in PFC is dynamic, even while the contents of WM remain stably represented. Here, we explored the neural dynamics in PFC during a memory-guided saccade task. We found evidence for dynamic population coding in various task epochs, despite striking stability in the neural representational geometry of WM. Furthermore, we identified two distinct cellular mechanisms that contribute to dynamic population coding.

## Introduction

Working memory (WM) provides the functional backbone to high-level flexible behavior. WM frees action from direct stimulus dependency, allowing information to be integrated over time for generating complex behaviors based on longer-term goals and contextual contingencies. Prefrontal cortex (PFC) is crucial for WM (Goldman-Rakic, 1987), yet the neurophysiological mechanisms that maintain information in PFC circuitry remain poorly understood.

According to persistent activity models of working memory, task-relevant information is maintained by keeping the corresponding neural representations active over memory delay periods through persistent neuronal firing (Curtis and D'Esposito, 2003; Funahashi, 2015; Riley and Constantinidis, 2016). A rich history of neurophysiological research has cataloged evidence for such persistent delay-period activity in prefrontal cortex (Fuster and Alexander, 1971; Kubota and Niki, 1971; Compte et al., 2000).

However, several lines of evidence complicate the persistent activity account (Barak and Tsodyks, 2014; Sreenivasan et al., 2014; Stokes, 2015). Persistently elevated, memory-specific delay-period activity turns out to be the exception for prefrontal cortex neurons, rather than the rule (Brody et al., 2003). Furthermore, neural activity increases toward the end of a fixed-duration delay period (Watanabe and Funahashi, 2007) and disappears altogether during simultaneous performance of an attentional task (Watanabe and Funahashi, 2014), thus highlighting the dependence of persistent spiking activity on attention and/or response expectation. Decoupling of sustained delay-period activity and the cognitive persistence of WM has also been observed during the performance of other WM tasks (Shafi et al., 2007; Barak et al., 2010). These studies, together with recent human neuroimaging studies (Riggall and Postle, 2012), suggest that plural (i.e., nonpersistent activity-based) mechanisms for WM maintenance can be observed in a wide variety of task contexts.

Accumulating neurophysiological evidence suggests an important role for dynamic population coding in the stable maintenance of WM in PFC (Meyers et al., 2008, 2012; Barak et al., 2010; Stokes et al., 2013). These studies have demonstrated that population-level activity patterns in PFC vary at the millisecond timescale during delayed match-to-category tasks (Meyers et al., 2008) and delayed paired-associate tasks (Stokes et al., 2013), and that such a dynamic code can be flexibly acquired after training on a given task (Meyers et al., 2012). These dynamics could reflect time-varying processes associated with encoding WM into an “activity-silent” neural state (Stokes, 2015). In particular, computational models demonstrate that WM can be maintained in an activity-silent form by relying on known mechanisms of short-term synaptic plasticity (Hempel et al., 2000; Mongillo et al., 2008; Sugase-Miyamoto et al., 2008; Lundqvist et al., 2016; Mi et al., 2017).

It is as yet unclear whether dynamic coding previously observed in PFC reflects task-specific cognitive transformations (i.e., categorization in the study by Meyers et al., 2008; recall of the associated pair in the study by Stokes et al., 2013) or forms a more general hallmark of WM. Moreover, very little is currently known of the underlying mechanisms of dynamic coding. It is well established that cells within PFC have different onset latencies (Riley et al., 2016), which could give rise to population-level dynamics (Harvey et al., 2012). On the other hand, dynamic population coding could also be mediated by dynamically switching selectivity within neurons (Sigala et al., 2008; Rigotti et al., 2013; Enel et al., 2016). To explore these possibilities, we examine the nature of PFC coding at the population level and single-cell level during the performance of variants of the memory-guided saccade (MGS) task. This task requires only a very simple transformation from stimulus location to saccade motor plan. For this reason, it has been particularly influential in the development of neural circuit models of WM (Compte et al., 2000; Wang, 2001), especially in the view that persistent delay activity is the primary substrate of WM maintenance (Constantinidis and Wang, 2004; Riley and Constantinidis, 2016).

To summarize our core results, across all experiments, we found consistent evidence that the PFC code for spatial location in WM consists of highly dynamic phases corresponding to cue processing and memory encoding, and a stable code during the later part of the memory delay, while the representational geometry remains stable throughout these dynamics and across all task epochs. This suggests that the mapping between neural activity pattern and memory content is not constant. We identified two mechanisms that underpin the observed dynamic-coding profiles. First, different neural subpopulations are involved in stimulus coding at different time points (dynamic subpopulation recruitment). Second, individual neurons have time-varying stimulus preferences (dynamic selectivity).

## Materials and Methods

### 

#### Subjects and apparatus

##### Experiment 1.

Data from this experiment have been analyzed for different research questions and reported previously (Watanabe and Funahashi, 2007). Two adult male macaques were used (monkey R: *Macaca mulatta*; weight, 8.5 kg; age, 11 years; monkey Z: *Macaca fuscata*; weight, 5.6 kg; age, 8 years). The monkeys were housed individually. The light/dark cycle was 13 h/11 h (light from 8:00 A.M. to 9:00 P.M.). Before starting the training of behavioral tasks, an eye coil and a stainless steel headpost were implanted in an aseptic surgical procedure, which has been described in detail previously (Watanabe et al., 2006). Following the completion of behavioral training, craniotomy was performed to make a small hole (20 mm in diameter) on the lateral surface of the prefrontal cortex. The position of the craniotomy was determined by structural MR images taken at the National Institute of Physiological Sciences, Japan. The stereotaxic coordinates of the center of the hole was 30.0 mm anterior to the interaural line and 15.0 mm lateral to the midline. A stainless steel recording chamber (20 mm in diameter; Narishige) was attached to the hole. During training and recording sessions, the monkey was seated in a primate chair in a dark sound-attenuated room with its head movement restricted by a headpost. The monkey faced a 21 inch CRT monitor (Flex Scan, Eizo) placed 40 cm away from the face of the monkey. Eye movements were monitored by the magnetic search coil technique. Control of behavioral tasks and data collection were accomplished using a TEMPO system (Reflective Computing).

##### Experiments 2 and 3.

Data from these experiments have been analyzed for different research questions and reported previously (Single Memory Task and Dual Memory Task, respectively, from Watanabe and Funahashi, 2014, 2015). We used two Japanese monkeys that were different from those used in Experiment 1 (monkey S: male; weight, 9.1 kg; age, 9 years; monkey A: female; weight, 5.5 kg; age, 6 years). The apparatus and surgical procedures were the same as those in Experiment 1, except that a lever (customized microswitch) was attached to the front wall of the monkey chair.

All experimental protocols were approved by the Animal Research Committee at the Graduate School of Human and Environmental Studies, Kyoto University, and were in full compliance with the guidelines of the Primate Research Institute, Kyoto University.

#### Behavioral paradigm

##### Experiment 1.

**Figure 1.**
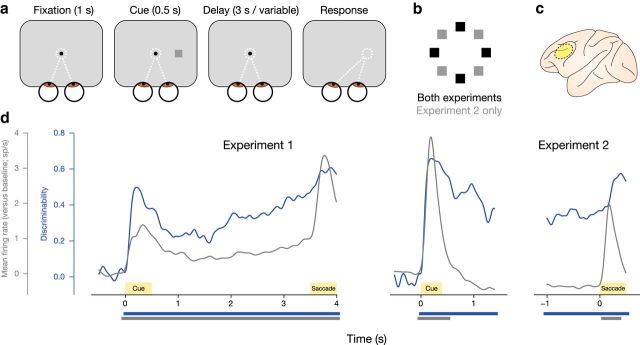
Overview of experimental paradigm and population-level pattern analyses. ***a***, Classical MGS task. Monkeys were trained to retain fixation and then make a saccade to a cued location after a fixed (Experiment 1) or variable (Experiment 2) delay. ***b***, Possible locations of memory cues. Black squares represent possible locations in both Experiments 1 and 2; locations depicted with gray squares were only present in Experiment 2. ***c***, Approximate location of neural recordings for all experiments, shown on a left hemisphere of macaque brain: lateral PFC. ***d***, Mean population firing rate (gray) and location discriminability of task conditions (blue) as a function of time for Experiments 1 and 2. Bars underneath the axes indicate significant changes from baseline.

We used a standard MGS task (Funahashi et al., 1989) with a fixed 3 s delay period. The temporal order of task events is shown in Figure 1*a*. The monkeys were required to make a memory-guided saccade after a 3 s delay to the location where a visual memory cue had been presented. Each trial began with the appearance of a fixation point (FP; a small white circle, 0.5° in visual angle) at the center of the monitor. After the monkey looked at the FP for 1 s, a visual memory cue (white circle, 1°) appeared for 500 ms (cue period) at one of four predetermined peripheral locations (0°, 90°, 180°, or 270° relative to the FP; 17° eccentricity). The location of the memory cue was randomized across trials. The monkey was required to maintain fixation at the FP until the end of the 3 s delay period. At the end of the delay period, the FP was extinguished and monkeys were required to make a saccade within 400 ms (response period) to the location where the memory cue had been presented. A drop of juice was given as a reward for a correct saccade.

##### Experiment 2.

We used a standard MGS task similar to that used in Experiment 1. However, there are two important differences: the length of the delay period was randomized across trials (0.5–8.1 s), and the location of memory cue presentation was selected from eight locations equally spaced (between 0° and 315° directions relative to the FP) on an imaginary circle (13° radius). Only trials with a memory delay duration of at least 1 s were included in the analyses. The memory cue was on the screen for 400 ms in this experiment.

##### Experiment 3.

This experiment was performed during the same recording sessions as in Experiment 2. The task used in this experiment consisted of two simultaneously performed cognitive tasks: an attention task and an MGS task (i.e., dual task). Monkeys were required to attend to one of three placeholders in the visual hemifield contralateral to the recording hemisphere and to keep a lever depressed. When the cued placeholder changed color, the monkey released the lever (the attention task component). During the delay period of this attention task, the MGS task was initiated by the presentation of the memory cue. The location of memory cue presentation was selected from five locations in the visual hemifield contralateral to the recording hemisphere (including two locations along the vertical meridian relative to the FP). For full details of the behavioral tasks in Experiments 2 and 3, refer to the study by Watanabe and Funahashi (2014).

#### Data collection

In all three of the experiments, we recorded single-neuron activity from the cortex within and surrounding the principal sulcus using glass-coated elgiloy microelectrodes (0.5–2.0 MΩ at 1 kHz). Electrodes were advanced by a hydraulic microdrive (MO-95, Narishige). Raw signals were filtered (300 Hz to 10 kHz) and amplified (DAM80, WPI). Single-neuron activity was isolated on-line using a window discriminator (DIS-1, BAK Electronics) and monitored continuously by a loudspeaker and two oscilloscopes (SS-7802, IWATSU). The monkeys performed the MGS task while the electrode was advanced into the cortex. We searched for well isolable neuronal activity that exhibited location selectivity in any of the task epochs by audiovisual monitoring of acquired signals. If such activity was not found, we recorded any well isolable neuronal activity that was encountered during the search. Time stamps of action potentials and behavioral events were stored in magnetic media by TEMPO for off-line analyses. In Experiments 2 and 3 only, spike wave forms and raw signals were digitized at 20 kHz (PowerLab 8/35, AD Instruments) and stored using custom software (Chart, AD Instruments). In Experiment 1, neural recording was performed predominantly from the dorsolateral portion of the PFC, while in Experiments 2 and 3, approximately three-fourths of recording sites were located in the dorsolateral PFC, with the rest located in the more ventral subregion of the lateral PFC. To exclude neurons recorded in the frontal eye field (FEF), intracortical microstimulations (22 biphasic pulses, 0.2 ms duration at 333 Hz, ≤150 μA) were applied through microelectrodes. When eye movements were elicited at <50 μA, the site was considered to be in the low-threshold FEF (Bruce et al., 1985), and data obtained at these sites were excluded from the database.

#### Data selection and preprocessing

Neurophysiological data were analyzed for successfully completed trials only. We excluded neurons that exhibited <500 spikes in a session from the database. The number of trials analyzed per neuron was 48 ± 13 (mean ± SD) in Experiment 1, 99 ± 21 in Experiment 2, and 131 ± 36 in Experiment 3. Binary spike trains were converted to spike rates by convolution of a Gaussian kernel with an SD of 50 ms. After the convolution, data were downsampled to 100 Hz. All data analysis was implemented in Python using the NumPy (van der Walt et al., 2011), SciPy (Jones et al., 2001), Matplotlib (Hunter, 2007), and Scikit-learn (Pedregosa et al., 2011) libraries, as well as custom-written code.

#### Statistical testing

Unless otherwise indicated, all statistical tests were performed using cluster-based nonparametric permutation tests (Maris and Oostenveld, 2007). This standard testing approach leverages the inherent correlation between consecutive time points (or time point pairs, in cross-temporal analyses) to control for multiple comparisons. Because of this inherent correlation, any true effect should be detected in several consecutive time points (or time point pairs), while any false-positive result is just as likely to show up in an isolated time point as it is in clusters of neighbors. Therefore, the cluster-based permutation tests compare only the maximum observed cluster of effects to a randomization-based distribution of such clusters under the null hypothesis, thus controlling for multiple comparisons while retaining statistical sensitivity.

Specifically, test statistics (e.g., *F* value, correlation coefficient, or raw difference) were computed for every time point or pair of time points. This was done both for the observed data and for each of 1000 permutations of randomly shuffled memory cue locations (for selectivity analysis) or time points (for analyses of significant changes through time). At every time point or pair of time points, candidate clusters were identified by comparing the observed test statistic to the 95th percentile of the permutation distribution. Neighboring time points (per pairs) exceeding this threshold were grouped together as one cluster candidate. We computed the maximum summed cluster test statistic for the observed data and compared this to the distribution of the maximum summed cluster test statistic across the permutations. This comparison yields the *p* value of the test (i.e., if the observed maximum summed cluster test statistic exceeds the 95th percentile of the permutation distribution of the maximum summed cluster test statistic, the difference in conditions is deemed to be significant).

#### Time-specific and cross-temporal discriminability analyses

**Figure 2.**
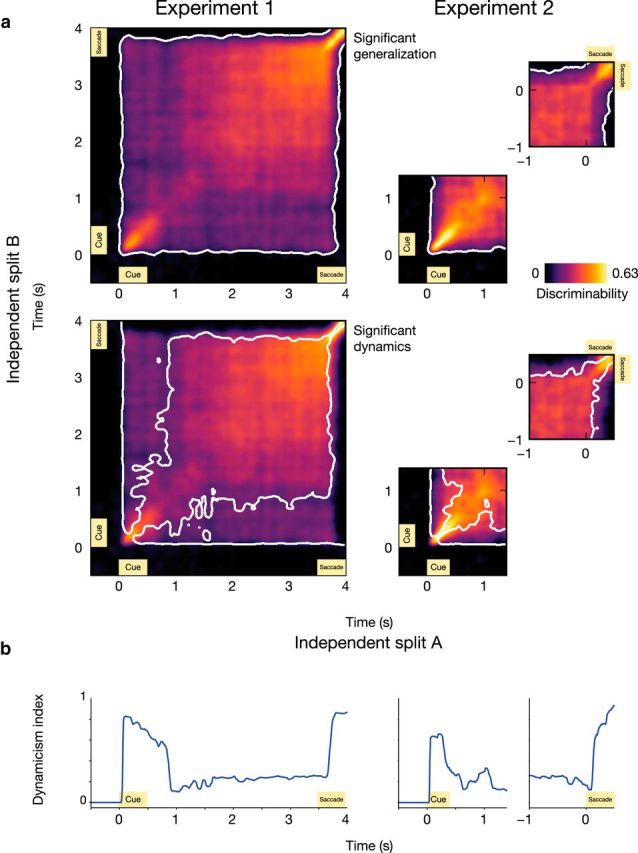
Cross-temporal discriminability analysis shows periods of dynamic and stable coding. ***a***, The cross-generalization discriminability score is color coded (i.e., the correlation of pairwise condition differences between all combinations of time points). White contours in the top plots indicate significant generalization; white contours in the lower plots indicate significant off-diagonal reduction (i.e., significant dynamic coding). ***b***, The dynamicism index provides an overview of the dynamic coding profile across time. Peaks in this plot are indicative of a strongly dynamic neural code, while valleys here correspond to plateaus of robust cross-temporal generalization.

Multivariate discriminability of WM contents within the PFC population activity was assessed using the analysis described in the study by Stokes et al. (2013), which lends itself well to the population of those non-simultaneously recorded (i.e., “pseudopopulation”), of which this dataset consists. We randomly assigned each trial to one of two independent data splits, *s* ∈ {*A*,*B*}. We then computed the mean activity *x̄* over all trials per split *N_s_*, per neuron *l*, per condition *k*:

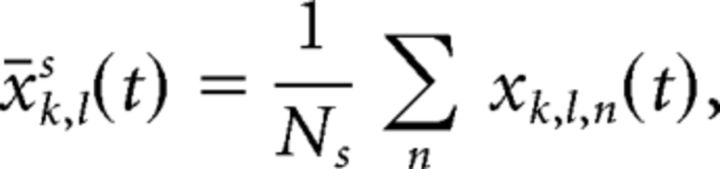
 where *x_k_*_,_*_l_*_,_*_n_*(*t*) is the firing rate in an individual trial. Then, for each independent split and each neuron, we computed the pairwise differences in activity between all possible pairs of conditions [a condition is a specific memory cue location; there are 6 (Experiment 1), 28 (Experiment 2), or 10 (Experiment 3) condition pairs]:

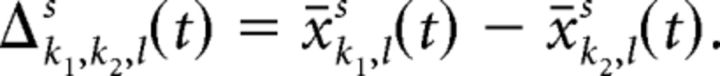
 The Pearson correlation of these pairwise differences across neurons between the two independent splits is a measure of the decodability of specific condition pairs from the PFC population, as follows:

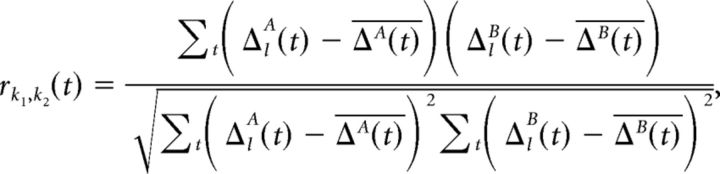
 where we dropped the *k* subscripts from Δ for clarity, and the overline denotes taking the mean over neurons. This metric quantifies to what extent the population-level pattern discriminating between two conditions is consistent between two splits of the data. If there is no such pattern, it is by definition not consistent between two splits, and thus the metric will be near zero. We averaged these condition pair-specific correlations using Fisher's *z*-transformation to obtain a single time-resolved discriminability measure, as follows:

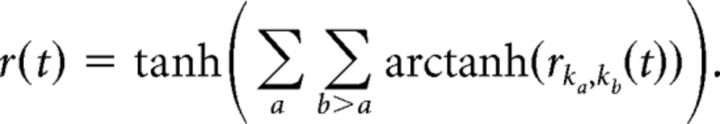
 The measure above is defined for each time point in a given task. It indicates the discriminability of memory cue location conditions at any particular time point (Fig. 1*d*) and is analogous to the ability of a classifier trained at time point *t*_1_ from data split *A* to decode the memory cue location condition in data split B at the same time point *t*_1_. It is straightforward to extend this definition to investigate cross-temporal decoding as well. For this, we computed the correlation of the pairwise differences at all time points *t*_1_ with all (same or other) time points *t*_2_, as follows:

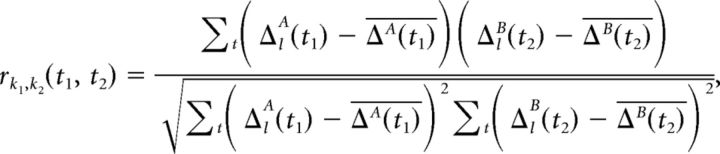


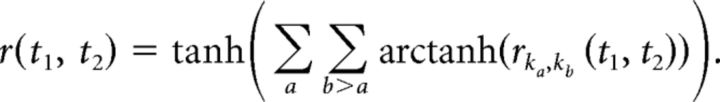
 The result serves as a measure of the cross-temporal discriminability of WM contents (memory cue location condition) from the PFC population activity (Fig. 2). Although this matrix is not mathematically symmetric, it is conceptually symmetric because the two independent data splits are randomly defined. Significance of discriminability was assessed by the cluster-based permutation test with randomly shuffled memory cue location conditions.

To test whether there is significant dynamic coding at a particular time point, we asked whether there was evidence for significant off-diagonal reduction in discriminability in the matrix *r*(*t*_1_, *t*_2_) relative to the corresponding on-diagonal values. Specifically, we computed the quantities *r*(*t*_1_, *t*_1_) − *r*(*t*_1_, *t*_2_) and *r*(*t*_2_, *t*_2_) − *r*(*t*_1_, *t*_2_) and tested whether these values were significantly greater than zero using the cluster-based permutation test in which the null distribution was estimated by randomly shuffling on-diagonal versus off-diagonal time points. To satisfy our operationalization of dynamic coding, both these tests had to yield a significant effect. In other words, the test of dynamic coding is equivalent to the following conjunction test:

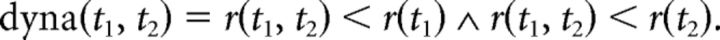
 It should be emphasized that the discriminability is defined using two independent data splits; therefore, a stronger on-diagonal than off-diagonal decoding performance is nontrivial (if the two data splits were nonindependent, this on-diagonal bias would be trivial).

For a convenient index of the amount of dynamic coding over time (Fig. 2*b*; also see Fig. 5*b*), we collapsed the binary significance matrix dyna(*t*_1_,*t*_2_) by averaging over the two time dimensions to yield what we call the dynamicism index (*di*), as follows:

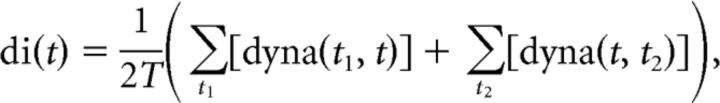
 where [*x*] denotes the Iverson bracket to yield 1 if *x* is true and 0 otherwise.

#### Analysis of single-neuron location selectivity

**Figure 3.**
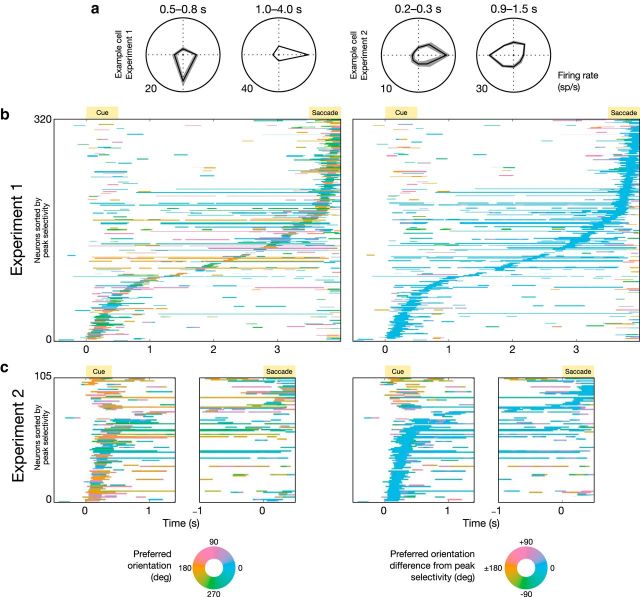
Single-neuron selectivity analysis reveals neurons with time-varying selectivity. ***a***, Two example neurons, showing a changing location preference over time (left: Experiment 1; right: Experiment 2). Time windows were determined by a cluster-based permutation test. Angles correspond to the cue location condition. Shaded area corresponds to the SEM. ***b***, ***c***, Color-coded location preference over time for neurons that showed significant task-related activity modulation for at least one time point. Left panels show the absolute location preference. Right panels show the circular difference between the preferred location of a neuron at any time point and that at the time point of peak selectivity. Neurons were sorted according to the time point of their peak location selectivity. ***a***, Experiment 1. ***b***, Experiment 2 (left, pericue period; right, periresponse period).

The influence of task conditions on single-neuron activity (Fig. 3) was analyzed by a standard one-way ANOVA. To correct for multiple comparisons, the resultant *F* statistics were subjected to the cluster-based permutation test described above. Note that the cluster-based permutation test conducted for each neuron controls for multiple comparisons across time, but not across neurons. Therefore, when interpreting the percentages of neurons showing significant selectivity (or significant change in selectivity), one should keep in mind that, by chance, 5% of neurons are expected to show significant selectivity (or significant change in selectivity).

To examine whether the location selectivity of each neuron changed over time, we computed the difference in single-neuron activity between each time point *t*_1_ and each (same or different) time point *t*_2_ and subjected this difference score to an ANOVA. We tested the resulting 2D matrix of *F* statistics for significance using the cluster-based permutation test, permuting time labels. A significant effect indicates that there was a change in location selectivity from one time point to another, which is analogous to an interaction effect between time and memory cue location condition. Since such an interaction effect can be also observed in a neuron that exhibits multiplicative gain in one time point relative to another (i.e., a neuron that is more responsive to one particular location at one time point than another but does not change its actual location tuning), we additionally required neurons to have an absolute angular difference in preferred location between *t*_1_ and *t*_2_ to be larger than one condition bin spacing (four location experiment, 90°; eight location experiment, 45°). Finally, the activity of the neuron was required to be significantly modulated by the location condition (the main effect of location in the ANOVA) at both *t*_1_ and *t*_2_. We imposed this requirement to exclude neurons that showed a main effect of location at *t*_1_ but not at *t*_2_ and vice versa, because these neurons simply lost their selectivity in one of the two time points (since just a main effect at *t*_1_ and no effect at *t*_2_ also satisfies the definition of an interaction).

We additionally computed a continuously varying estimate of the location preference of a neuron (Fig. 3, color scale). In accordance with previous work, one can view each data point as a vector in complex space, where the angle is given by the cue location of the current trial, and the magnitude is given by the firing rate of the neuron. The continuous preferred location across trials is given by the angle of the circular mean, as follows:

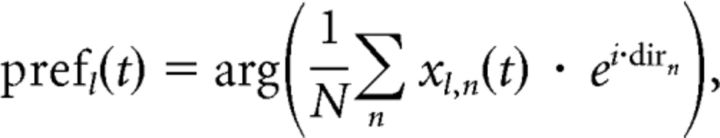
 where *x_l_*_,_*_n_*(*t*) is the activity of neuron *l* in trial *n* at time *t*, dir*_n_* is the (angular) direction of the memory cue on trial *n*, and arg denotes the complex argument (Takeda and Funahashi, 2004; Zar, 2014). To prevent a possible bias due to unequal trial numbers among conditions, we randomly removed trials until trial counts were equal among conditions before estimating this measure.

#### Simulation analysis: relative contributions of dynamic subpopulations and dynamic selectivity

We used a simulation approach to quantify which of the two observed phenomena, dynamic subpopulation recruitment or fluctuation of location selectivity in individual neurons across time, is a driving force underlying the dynamic coding we observed in various task epochs. The intuitive approach would be to exclude all neurons that significantly changed location selectivity across time (switching neurons) from the dataset and simply recalculate the dynamics. However, we note that simply removing the switching cells does not control for differences in time-specific selectivity associated with each cell. This could result in a bias toward reduced dynamic coding due to a lower diagonal of the cross-temporal generalization matrix.

To overcome this problem, we simulated two new neural populations based on the observed dataset, one without removing switching neurons and one with switching neurons removed. Specifically, we quantified the selectivity profile for each observed neuron *l* at each time point by computing the mean firing rate over trials per memory cue location condition *k*, as follows:

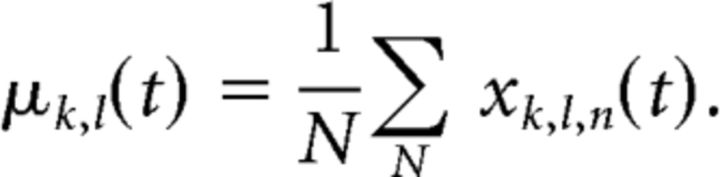
 This measures the expected response of a neuron in each memory cue location condition. Any changes across time in the condition-specific response pattern (i.e., how μ*_k_*_,_*_l_* varies as *k* varies) indicates a switch in location selectivity. Note that, by design, this is a much looser definition of selectivity switching than the statistical inference on what constitutes a switch than we used before; here we just want to capture any possible change in selectivity pattern.

To simulate the neural population with selectivity switches intact, we simply draw trials from these sample means, as follows:

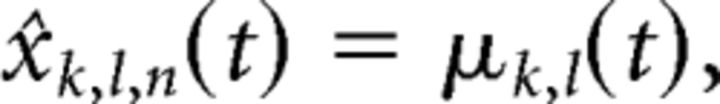
 which gives the simulated rate for trial *n*. Binary spike data were drawn from a Poisson process using this underlying rate. It should be noted that analyses on this simulated population with switches intact results in a direct approximation of the actually observed data, with quantitative variation due to the specific instantiation of the Poisson spiking model.

To simulate the population with switching selectivity removed, we set the selectivity profile at all time points to be identical to the time point *t*_peak_ at which the neuron had its maximum firing rate. Importantly, the shape of the condition-specific pattern was fixed across time, but the overall amplitude was not, to allow any dynamics due to time-varying amplitude to remain intact:

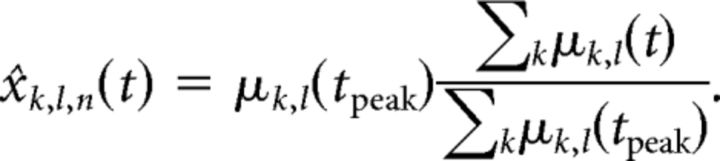
 Again, binary spike data were drawn from a Poisson process using this underlying rate.

**Figure 4.**
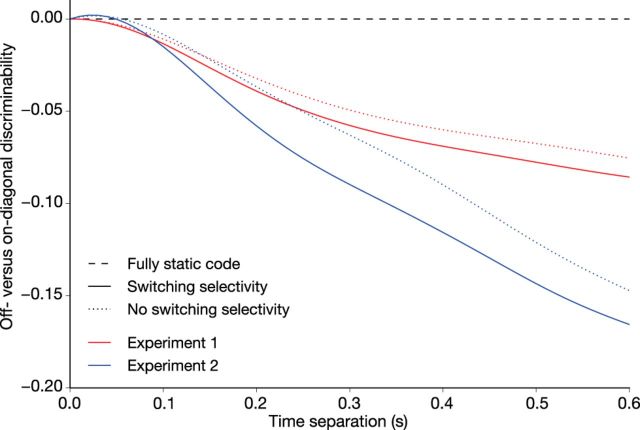
Relative contributions of changing selectivity and onset variability to dynamic coding. Shown on the *y*-axis is the degree of discriminability (as in Fig. 2*a*) on off-diagonal time points, which are expressed relative to the diagonal. The steepness in dropoff as a function of lag is an indication of the extent of population-wise dynamic coding. This analysis was performed on the whole trial and was not limited to any particular task epoch. Simulating a completely fixed selectivity reduces the extent of dynamic coding. The substantial residual dynamics can be attributed to neuronal onset variability.

We computed full cross-temporal discrimination matrices based on the two simulated populations (switches intact and switches removed) and computed the difference in off-diagonal versus on-diagonal coding as a function of time lag (Fig. 4). Time lags throughout the whole trial were used. The gradient of these functions is an indication for the amount of dynamic coding: a flat line indicates a fully static code and a steep gradient indicates a strongly dynamic code. The change in gradient for switches removed versus switches intact indicates the contribution of switching neurons to the observed dynamics, with any remaining dynamics attributable to consistent variation in neuronal onset latencies.

#### Analysis of multitask single neuron selectivity

To investigate the modulation of the activity of individual neurons by combinations and/or interactions of different task factors in Experiment 3 (see Fig. 6*c*), we performed a 3 × 5 ANOVA followed by cluster-corrected permutation tests on the three resulting *F* statistics (main effects of attention and WM factors, interaction effect). Analogous to the single-factor ANOVA that was used for the memory task performed alone, we also computed these statistics on the difference scores between all possible time-point combinations to assess whether significant changes across time could be identified.

## Results

We recorded single-unit spiking activity from multiple neurons (*n* = 698/139/101 for Experiments 1/2/3) in the lateral PFC (Fig. 1*c*) of four macaque monkeys, performing a total of three experiments (two monkeys participated in Experiment 1; two monkeys participated in both Experiments 2 and 3). All experiments used variants of the MGS task. Monkeys were presented with a visual memory cue at one of four (Experiment 1), eight (Experiment 2), or five (Experiment 3) possible peripheral locations distributed uniformly on an invisible circle around a central fixation spot (Fig. 1*a*,*b*). They were trained to keep the location of memory cue presentation in mind for a fixed delay period (Experiment 1, 3 s) or a variable delay period (Experiments 2 and 3, 0.5–8.1 s; only delays ≥1 s analyzed). After the termination of the delay period, the monkey was prompted to make a saccade to the remembered location by the disappearance of the fixation spot (go signal). For full experimental details, refer to the Materials and Methods section and our previous publications on the same datasets (Watanabe et al., 2006; Watanabe and Funahashi, 2007, 2014). The majority of the results presented in this report focus on Experiments 1 and 2, with results for Experiment 3 presented at the end of the Results section.

### Temporal profile of working memory discriminability

In both Experiments 1 and 2, we observed significantly increased population activity (averaged over memory cue location conditions and neurons) during the presentation of the memory cue (Fig. 1*d*, gray traces). For Experiment 1, this elevation lasted throughout the (fixed-duration) delay period and peaked during saccade execution (cluster-based permutation test; *p* < 0.001). For Experiment 2, the firing rate returned to baseline shortly after the offset of the memory cue and remained at baseline levels throughout the (variable-length) delay period, only rising again during saccade execution (*p* = 0.008). This difference in firing rate elevation between Experiment 1 (fixed delay) and Experiment 2 (variable delay) is potentially due to differences in the timing of the go signal. In Experiment 1, the go signal was predictable and the monkey was able to anticipate its occurrence, whereas the timing of the go signal was unpredictable in Experiment 2.

To investigate the involvement of the PFC neural population in the coding of WM contents, we used a variant of a multivariate analysis method previously developed for population-level analysis of spiking activity data (Stokes et al., 2013). Briefly, we split the observed trials into two independent halves and computed the average firing rate per neuron, per condition, for each of these halves. Then, we computed the differences in firing rate between all possible condition pairs. The correlation of these pairwise differences across neurons between the two independent splits provides a continuous, bounded, unbiased measure for how reliable the PFC population can discriminate between the task conditions. This correlation is analogous to the performance of a linear nearest-neighbor classifier trained on split A and tested on split B (Haxby et al., 2014). For both Experiments 1 and 2, we observed significantly elevated discriminability throughout the cue and delay periods (Fig. 1*d*, blue traces; both *p* < 0.001). In Experiment 2, this period of high discriminability coincides with a time window where the firing rate is predominantly at baseline levels, thus confirming the ability of PFC neurons to represent WM-related information in the population-level response, despite low overall levels of activity.

In Experiment 1, we additionally noted that the discriminability reaches a local peak during the cue period, after which it falls into a distinct lull. Hereafter, the discriminability increases again throughout the delay period toward the time of the saccadic response. This pattern of “ramping” delay-period activity has previously been associated with the preparation for expected response demands (Barak et al., 2010) and suggests that delay-period activity can be flexibly modulated as a function of current task relevance, as opposed to it being a necessary precondition for maintenance per se.

### PFC representation generalizes over time, yet shows clearly dynamic epochs

The discriminability analysis shown in Figure 1*d* can be extended to analyze across-time discriminability. The cross-temporal extension of the generalization test provides an important index of the time specificity of discriminative patterns (King and Dehaene, 2014). If the underlying representation is stationary, it should not matter whether a classifier is trained on one particular time point during the coding epoch and then tested on another. However, if the discriminative representation is dynamic, then decoding should be optimal only when comparing neural patterns between two time points very close to each other.

To distinguish between these two scenarios, we correlated the pattern of pairwise condition differences at each time point *t*_1_ with the pattern at every (other or same) time point *t*_2_. The diagonal of the resulting two-dimensional matrix provides a time course of decodable information and is simply the time-specific discriminability that was depicted in Figure 1*d*. Significant off-diagonal elevation in this matrix is evidence for a neural population code that generalizes over time. We observed significant cross-temporal generalization of the neural population code in both Experiments 1 and 2 (both *p* < 0.001; Fig. 2*a*, top row). In Experiment 1, following a dip in discriminability at ∼0.5–1.0 s after cue offset, there was a clear “ramp-up” of activity (and generalizability) toward the timing of saccade execution, which occurred at ∼3.5 s relative to the timing of cue offset.

To test for the presence of dynamic population coding, we examined whether off-diagonal elements were significantly reduced with respect to the corresponding values along the diagonal. If so, we can conclude that the code for WM content changed significantly over time.

We observed a significant off-diagonal reduction in cross-temporal generalization and, hence, significant dynamic population coding during and following the cue period in both Experiments 1 and 2 (Fig. 2*a*, bottom row). Additionally, we observed significant dynamic coding during the response period of Experiment 1. The delay period in both experiments is characterized by a “plateau” of robust cross-temporal generalization, starting at ∼1 s after cue onset for Experiment 1 and at ∼800 ms after cue onset for Experiment 2. (Note that the data going into the response-locked analysis for Experiment 2 is temporally jittered with respect to cue onset; thus only time-general decoding will show up in this plot.) These epochs of dynamic coding are clearly identified when we express the level of coding dynamics as a time-varying scalar quantity, referred to as the dynamicism index (Fig. 2*b*; see Materials and Methods).

### Factors contributing to dynamic coding: changing neuronal selectivity

The evidence for dynamic coding reported above indicates a changing neural code for WM content over time. Such a changing neural code could indicate either that neurons change their location preference over time (Sigala et al., 2008; Rigotti et al., 2013; Enel et al., 2016) or, alternatively, that a different subpopulation of neurons is involved in the coding of memory cue location at different time points (yet with each neuron having a unique and stable location preference;). We find evidence for both of these phenomena.

We computed the magnitude of location selectivity of single neurons by means of a cluster-corrected test based on *F* statistics derived from one-way ANOVA. Then, we sorted all location-selective neurons (324 of 698 neurons for Experiment 1) according to the time of their peak *F* statistic, and computed the preferred direction of each neuron (see Materials and Methods for details). The results of this analysis for Experiment 1 are shown in Figure 3*b* (Fig. 3*a*, example neuron). A clear separation is visible between neurons predominantly active during the cue period (presumably reflecting sensory processing and encoding into WM; Rainer et al., 1999), those predominantly active during the response period, and those predominantly active during the delay period. When using a color code relative to the peak preferred direction of the neurons (Fig. 3*b*, right), any changes in selectivity become apparent. Within the dynamic time period corresponding to cue presentation (500 ms), a small number of location-selective neurons [8 of 136 neurons (6%)] showed a significant change in location selectivity (cluster-corrected significant interaction between time and task condition). This proportion barely exceeds the amount expected by chance, given α = 0.05. After analyzing the full trial, we found substantially more neurons that significantly changed their preferred location between different trial epochs. Of 324 location-selective neurons, 83 (26%) displayed significant time-varying location selectivity at any point in the trial. Focusing only on switches within the delay interval itself, we again find a negligible proportion of switches [10 of 189 switches (5%)], thus indicating that the switches in selectivity observed in Experiment 1 are primarily due to switches between different epochs of the task (in line with previous reports; Jun et al., 2010).

For Experiment 2, a significant proportion of neurons [24 of 92 neurons (26%)] displayed a significant change in location selectivity during cue presentation, while 37 of 100 neurons (37%) that exhibited significant location selectivity displayed significant time-varying selectivity on longer timescales following the cue (Fig. 3*c*). In Experiment 2, 13 of 73 neurons (18%) displayed significant changes in location selectivity within the delay period, although it should be noted that these changes occurred primarily during the early part (first 0.6 s) of the delay period (see Fig. 6*d*, cumulative percentage over time). The higher proportion of neurons that significantly changed location selectivity over time (switching neurons) in Experiment 2 compared with Experiment 1 could be explained by task differences. Experiment 2 involved a larger number of memory cue location conditions (eight) than Experiment 1 (four). Thus, there are more opportunities to detect a change in location preference for neurons recorded during Experiment 2 than for those recorded during Experiment 1.

### Factors contributing to dynamic coding: dynamic onset cascade

Although individual neurons showed significantly changing location selectivity both across different trial epochs and within the cue period, the plots of location selectivity relative to the preferred direction of each individual neuron (Fig. 3*b*,*c*) also show variation in the peak engagement time of individual neurons. Thus, as mentioned above, another factor that contributes to dynamic coding could be that different neurons become active at different points in time (Riley et al., 2016).

To test this formally, we computed the timing of the peak firing rate and the peak location selectivity for every neuron in two independent splits of the data (limiting ourselves to those neurons that were active during the cue epoch). We found a strong and significant correlation between these two independent splits in both the peak firing times (Experiment 1: Spearman ρ = 0.66; *p* = 1.2 × 10^−18^; Experiment 2: ρ = 0.70; *p* = 1.2 × 10^−16^) and the times of strongest location selectivity (Experiment 1: ρ = 0.47; *p* = 6.7 × 10^−5^; Experiment 2: ρ = 0.62; *p* = 1.5 × 10^−9^), suggesting that the order of neuronal firing and selectivity is preserved from trial to trial and is therefore a genuine property of the neural population.

We next sought to determine the relative contributions of changes in location selectivity and differences in neuronal onset latencies to population-level dynamic coding. We performed a simulation analysis to explicitly quantify the relative contribution of changes in neuronal selectivity to cross-temporal generalization. First, we parameterized the changes in location selectivity in the observed dataset and simulated trials drawn from that parameterization. We then performed the same analyses as before to recover the reference level of time specificity in the simulated PFC population. Next, we manually constrained each neuron to a single selectivity throughout the trial. This removes qualitative, but not quantitative, differences over time (i.e., any variation in onset latencies is preserved; for details, see Materials and Methods). Results for this analysis are shown in Figure 4. The gradient of the off-diagonal drop-off curve is a measure of dynamic coding. While it is clear that the presence of switching neurons contributes to dynamic coding (i.e., the curves become less steep in the absence of switches), the substantial remaining time dependency can be attributed to systematic differences in neuronal onset latencies.

Together, the observed time-varying location selectivity (Fig. 3) and the consistent heterogeneity of onset latencies for different neurons lead us to conclude that it is likely a combination of dynamic selectivity and dynamic subpopulation recruitment that leads to the dynamic population code observed in PFC during WM coding.

### Representational space is stable despite a dynamic population code

The analyses presented so far have focused on the characteristics of the neuronal population code during WM encoding and maintenance: we found a dynamic coding “ridge” during memory encoding followed by a stable coding plateau characterized by cross-temporal generalization during the later delay period. Yet, despite these dynamics, the monkey somehow maintains a stable representation in working memory, as is evident from its successful performance of the task. Thus, in the following section, we examine how the stability of the mental representation of memory cue location is maintained during population-level dynamics.

**Figure 5.**
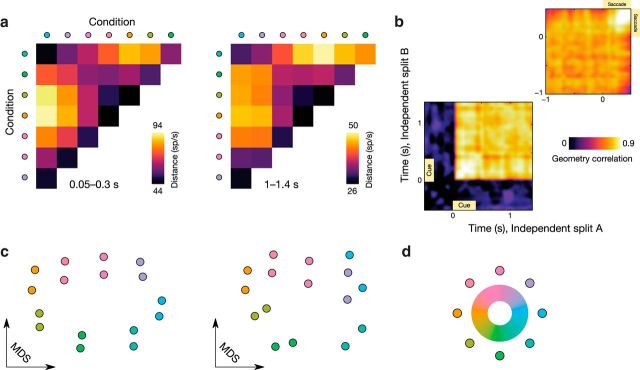
RSA reveals a stable representational geometry. ***a***, Pairwise Euclidean distances in neural space between all pairs of location conditions (Experiment 2). These pairwise distances are highly preserved between the cue interval (left) and the delay period (right). ***b***, Cross-temporal correlation between distance matrices such as those shown in ***a***, computed for all time points individually (in two independent data splits). ***c***, MDS plots for the distance matrices shown in ***a***. Two dots with identical color correspond to the two splits of the data. ***d***, Possible locations of memory cues (color coded).

For this, we focus on Experiment 2, because eight different cue locations (as opposed to four in Experiment 1) allow a detailed view on the representational geometry of the recorded PFC population. The Euclidean distances in neural space (i.e., the space spanned by the activity levels of all neurons) between all possible location–condition pairs describe this representational geometry; Kriegeskorte et al., 2008; Kriegeskorte and Kievit, 2013). We computed these distances for the following two time windows for Experiment 2: the early cue period (0.05–0.3 s) and the delay period (1–1.4 s). The pairwise distance matrices are displayed in Figure 5*a*. We find that, even though the underlying population code is dynamic, the representational state is highly stable (Pearson correlation of distance matrices: *r* = 0.91, *p* = 4 × 10^−11^). This is also borne out by the multidimensional scaling (MDS; Borg and Groenen, 1997) plots based on these distance matrices (Fig. 5*c*). During the cue period, the MDS plot almost exactly mirrors the physical distribution of cue locations (Fig. 5*d*). During the delay period, the pattern is somewhat less clear cut but is still strikingly consistent.

To investigate the potential changes in representational geometry over time in more detail, we computed the condition–pairwise Euclidean distance matrix for each time point individually in two independent splits of the data. Next, we correlated these distance matrices across all time points. Results for this analysis are shown in Figure 5*b*, where a clear plateau of cross-temporal generalization of the pairwise condition distances can be observed. Thus, even though the PFC code is, as demonstrated in the previous sections, highly dynamic in nature, the representational geometry is remarkably stable throughout the trial.

### Simultaneous performance of a competing task does not abolish dynamic coding and reveals neurons with complex mixed selectivity

**Figure 6.**
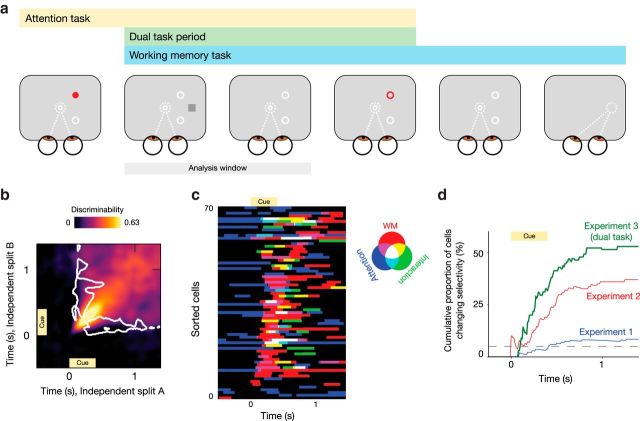
Dynamic coding is preserved during a more complex dual-task scenario. ***a***, Task structure for Experiment 3. Monkeys were engaged in an attention task (red dot and circle) while the working memory task was initiated (as before, with a location-specific cue; gray square). ***b***, Cross-temporal generalization matrix for the memory cue period in Experiment 3. White contours indicate significant off-diagonal reduction and thus dynamic coding. ***c***, Results of a per-neuron (cluster-corrected) 3 × 5 ANOVA for the attention (three levels) and memory (five levels) conditions. Significant effects are color coded using additive color mixing. Neurons with significant selectivity to at least one factor are sorted according to the time point of their peak selectivity to the memory condition. Note the high fraction of neurons responsive to combinations of task factors. ***d***, Cumulative proportion of neurons changing condition selectivity, as a function of time. The number of neurons that show a significant change in condition selectivity up to any particular time point, expressed as a proportion of neurons that have any significant selectivity at all, up to that time point. Note that this is a ratio between two cumulative quantities; thus, negative steps are possible. The dotted line indicates the percentage of neurons (5%) expected to satisfy the criterion for changing selectivity by chance. The dual task is plotted in green, while the results of the two single-task experiments are plotted in blue and red, for comparison. A richer task structure reveals a more dynamic view of single-neuron PFC selectivity.

The two monkeys participating in Experiment 2 also participated in Experiment 3 (Watanabe and Funahashi, 2014). During this experiment, monkeys were concurrently engaged in two tasks (i.e., dual-task experiment). They were presented with a visual cue for an attention task at the beginning of a trial, while they were depressing a lever. After an attentional delay, the to-be-attended stimulus changed its color, prompting the monkeys to release this lever. The memory-guided saccade task was initiated (i.e., memory cue presented) during the attentional delay. After the dual-task demand was resolved (i.e., after lever release), the task proceeded as the normal MGS task with a memory-guided saccade after some delay (Fig. 6*a*).

Replicating our previous results, we observed evidence for dynamic coding (followed by significant cross-temporal generalization) during and just after the presentation of the memory cue in Experiment 3 (Fig. 6*b*).

The reported analyses for Experiments 1 and 2 focused on the selectivity of the neural population for the WM condition. However, it is becoming increasingly clear that individual PFC neurons often display a very high-dimensional selectivity (i.e., they tend to respond to complex mixtures of various task parameters; Asaad et al., 1998; Mansouri et al., 2006; Rigotti et al., 2013). Therefore, any analysis of selectivity to a single task condition might underestimate the amount of changing neuronal selectivity. The data for Experiment 3 allowed us to assess whether this was the case for the present analyses.

Since the monkeys were involved in two tasks at the same time, we could now analyze the contribution of both the attention and the memory factor to the neuronal code. We analyzed the activity of each neuron using a two-way 3 × 5 ANOVA (cluster corrected). Results for this analysis are shown in Figure 6*c*. The different colors indicate significant effects of (combinations of) the task factors and their interaction. Clearly visible is an onset cascade of selectivity to the memory cue (red/magenta/yellow/white) following cue onset (*t* = 0 s). This is preceded by selectivity only to the attentional task (blue). Interestingly, shortly following cue onset, many neurons start to become tuned to combinations of and/or interactions between the two task factors (any color but pure red or blue). After analyzing the cumulative proportion of neurons with significantly changing selectivity (Fig. 6*d*, green curve), we find that this fraction is indeed higher when multiple task factors can be taken into account. It should be noted that this is a different type of switching selectivity than those between different angles, as shown in Experiments 1 and 2. However, this result underlines the idea that typical (low-dimensional) experiments tend to underestimate the dimensionality of the PFC population code.

## Discussion

We have provided evidence from three separate experiments for the existence of dynamic population coding for WM contents in the lateral PFC. Cue processing, memory encoding, and motor execution were highly dynamic, while the later part of the memory delay interval was characterized by robust cross-temporal generalization. Importantly, we observed this dynamic neural code while subjects were performing a classic memory-guided saccade task that has previously been influential in developing models of persistent activity-mediated WM. We identified two phenomena that could explain dynamic population coding: a rapid neural cascade during cue processing; and changing neuronal selectivity over longer timescales. The representational space of the PFC population remained stable during periods of high dynamic coding, indicating a flexible mapping between WM representation and neural code.

### Dynamic coding in working memory

The main result of this series of analyses is that population coding during both the processing of WM cues, and the early part of the subsequent maintenance period, is highly dynamic. Previous studies have highlighted the importance of neural dynamics in population coding (Romo et al., 1999; Brody et al., 2003; Mazor and Laurent, 2005; Meyers et al., 2008; Crowe et al., 2010; Harvey et al., 2012; Stokes et al., 2013; Astrand et al., 2015; Vergara et al., 2016), which might constitute a general property of neural processing (Buonomano and Maass, 2009; Buzsáki, 2010).

We here show dynamic coding during various epochs of a task in which successful performance is classically associated with stable, sustained maintenance of task-related neuronal activity. To date, the majority of evidence for dynamic coding has been found during relatively complex tasks that presumably engage a number of cognitive transformations (Meyers et al., 2008; Stokes et al., 2013) and/or distinct cognitive epochs (Sigala et al., 2008; Barak et al., 2010). Arguably, transforming stimulus identity into a category-level representation (Meyers et al., 2008) or a cue stimulus to a previously learned association (Stokes et al., 2013) entails distinct cognitive episodes with distinct coding patterns (Sigala et al., 2008). Cognitive transitions are minimal in the MGS task studied here. The subject must simply retain the location of the initial cue and generate the appropriate saccade at the end of the trial. The simplicity of the MGS task has contributed to the role of this paradigm in shaping current models of persistent stable activity (Curtis and D'Esposito, 2003; Funahashi, 2015).

Nevertheless, using population-level analyses, we observe the hallmark of dynamic coding during several epochs of such a simple cognitive task. Presumably, some of these dynamics could reflect transformation from a stimulus to a saccade motor plan. However, it is evident from Experiment 1 that motor preparation is unlikely to fully explain the dynamic coding observed at the beginning of the trial. Specifically, population coding during the most dynamic 1 s at the start of the trial differs significantly from the population code during the late delay and subsequent response periods. Neural activity in the response period is most likely to reflect motor execution, which is thus distinct from the neural code observed early in the trial. We believe the most likely explanation for the dynamics in the early part of the delay period is the transformation from transient sensory input into a stable working memory representation. This transformation will consist in a high-energy dynamic trajectory through neural state space, as predicted by a dynamic coding model of working memory (Stokes, 2015), while the WM representation itself should be low energy and stationary (potentially because the primary substrate is a “hidden” state of the network; e.g., synaptic connectivity). These predictions are in accordance with our results. Critically, we now relate these population-level dynamics to underlying single-cell dynamics.

### Stable representational geometry

The parametric memory space used in the eight-location memory-guided saccade task allowed us to go beyond the neural dynamics of WM coding and explore the representational geometry of the mental representation. This approach leverages the relative dissimilarity in cue locations to characterize the geometry of the representations in activity patterns. Representational similarity analysis (RSA; Kriegeskorte and Kievit, 2013) abstracts over the specific activity patterns to consider how different conditions relate to one another in “representational space.” These tools allow us to examine the relative configuration in state space regardless of the specific coordinates, which is particularly important for testing the representational structure in dynamic population coding (Cichy et al., 2014).

The memory-guided saccade task was chosen for the current analyses of dynamic coding specifically because of the inherent cognitive stability (i.e., keep one location in mind and saccade to that exact location) associated with this simple paradigm. The representational similarity analyses confirmed the expected stationarity—the representational geometry was very stable, despite the rapid dynamics in the underlying patterns of population activity. The PFC population represents the same information throughout the task but uses different discriminative patterns over time.

### Active versus silent (hidden) maintenance of WM

Mongillo and colleagues have proposed a synaptic model of WM (Mongillo et al., 2008; Barak and Tsodyks, 2014) in which memories are stored via short-term synaptic plasticity (which is especially prominent in prefrontal cortex; Hempel et al., 2000) without concomitantly elevated firing rates. Memories can be effectively read out via uniform input that drives activity through the memory-conditioned network to generate a memory-specific output response. The important principle is that previous stimulation history can be recovered from the hidden state of a network (Nikolić et al., 2009; Wolff et al., 2015, 2017; Rose et al., 2016), allowing for an energy-efficient model of short-term memory that does not rely on continuous maintenance of stable high-energy activity states (Stokes, 2015).

The MGS task is a classic paradigm used to study delay-period activity (Funahashi et al., 1989; Goldman-Rakic, 1995) and has been extremely influential in developing models of the persistent maintenance of WM contents (Compte et al., 2000; Wang, 2001). However, the singular nature of the task could have overemphasized the mnemonic role of persistent delay activity. Memory-specific persistent delay activity could reflect the focus of attention to the most relevant item in WM (Lewis-Peacock et al., 2012; Riggall and Postle, 2012), which could benefit efficient readout but might not be strictly necessary for WM maintenance. This account would help to explain the ramping delay activity we observed in Experiment 1 as well as previous reports indicating a reactivation of WM-related firing patterns when the contents of WM become necessary to guide oculomotor behavior (Watanabe and Funahashi, 2014). It should also be noted that, under the synaptic WM hypothesis, WM conditions might still be discriminable from firing rates during memory delays, as even spontaneous “background” activity should be patterned according to the current hidden state. Indeed, in the present study we find (cross-temporally stable) discriminability of WM conditions even when mean activity was not significantly different from baseline levels (Experiment 2; Fig. 1*d*). Finally, it is also important to note that persistent activity-based models do not necessarily predict large overall changes in firing across the population; therefore, the current results do not directly adjudicate between persistent activity models and activity-silent working memory.

### Dynamic selectivity, dynamic subpopulations, and the neural null space

The dynamic code we report was partly explained by different neurons having different time courses of contribution to task-related activity. Importantly, these neuronal timing differences were consistent between independent splits of the data, thus confirming the existence of specific cell latencies in PFC (Riley et al., 2016), which is consistent with a neuronal involvement “cascade” (Harvey et al., 2012). Furthermore, we found evidence for a significant proportion of neurons changing their selectivity throughout the trial, particularly between different task epochs. This finding further corroborates the dynamic nature of the PFC code and provides a conceptual link between population-level and single-unit analyses (Rigotti et al., 2013).

Neuronal dynamic selectivity in particular and, to a lesser extent, the existence of dynamically active subpopulations have important consequences for how a downstream region might “read out” the representational content of a (PFC) population. Although a static (i.e., time-constant weights throughout the entire trial) linear classifier could in principle discriminate some relevant information (as evidenced by the presence of cross-temporal generalization across all task epochs; Fig. 2*a*), the observed dynamics mean that this readout will be suboptimal. Instead, the optimal readout will be different for different task epochs. If a downstream region is interested in the information only during one particular epoch, then it could use readout weights optimized for those time points and thereby “tune out” to the information at other time points. The only period of the trial throughout which a static classifier should be able to decode WM contents as efficiently as a dynamic one is the later part of the delay period, because this is where we observed a clear plateau of cross-temporal generalization of the neural code without any dynamic ridges. This is in line with recent reports of a “mnemonic subspace” constructed by decomposing time-averaged neural activity that captures a large proportion of stimulus variance throughout WM delays (Murray et al., 2017).

In the motor domain, recent research shows that preparatory cortical activity preceding a movement is likely largely confined to the “null space” of the projection from cortex to muscle (Kaufman et al., 2014). That is, preparatory activity lies in those parts of neural state space that lead to little or no activity in the downstream muscles. This occurs because the synaptic weights in the corticomuscular projections effectively cancel out any contributions from preparatory neural patterns that are mainly found in the delay period. While “blind” to preparatory activity, the downstream area can “see” activity meant to actually drive the muscles, as this will fall outside the null space of the projection. It has been shown that a similar mechanism is involved in corticocortical connections (Kaufman et al., 2014).

An intriguing speculation is that the changing code over time that we observed, along with projections with different null spaces, might facilitate such selective readouts, while allowing computations within the PFC itself (e.g., the transformation of a sensory code to a “prospective” code; Rainer et al., 1999) to take place without unduly disturbing downstream regions (i.e., such activity would lie within the null space of all downstream projections). It is worth noting that the concept of a neural projection null space has also been instrumental in the development of a computational model of constant representational content underpinned by a dynamic neural code (Druckmann and Chklovskii, 2012). Finally, it has been argued that neural dynamics along a projectional null space tend to result in neurons showing mismatches of selectivity among different task epochs (Churchland et al., 2010; Kaufman et al., 2014), which is in line with what we report here.
